# Photodynamic Antimicrobial Therapy Based on Conjugated Polymers

**DOI:** 10.3390/polym14173657

**Published:** 2022-09-03

**Authors:** Huanxiang Yuan, Zelin Li, Xiaoyu Wang, Ruilian Qi

**Affiliations:** 1 Department of Chemistry, College of Chemistry and Materials Engineering, Beijing Technology and Business University, Beijing 100048, China; 2 School of Materials Science and Engineering, University of Science and Technology Beijing, Beijing 100083, China

**Keywords:** conjugated polymer, photodynamic therapy, antimicrobial treatment

## Abstract

Pathogenic microorganisms have been a serious threat to human life and have become a public health problem of global concern. However, in the actual treatment there is a lack of efficient antimicrobial strategies which do not easily develop drug resistance; this can lead to inaccurate drug treatment that worsens the infection and even threatens life. With the emergence of a variety of drug-resistant bacteria and fungi, photodynamic therapy has gradually become one of the most promising treatment methods for drug-resistant bacteria infection; this is because it is controllable, non-invasive, and not prone to cause the development of drug resistance. Organic conjugated polymers that possess high fluorescence intensity, a large molar extinction coefficient, excellent light stability, an adjustable energy band, easy modification, good biocompatibility, and the ability to photosensitize oxygen to produce reactive oxygen species have been widely used in the fields of solar cells, highly sensitive detection systems, biological imaging, and anti-cancer and anti-microbial treatment. Photodynamic therapy is non-invasive and has high temporal and spatial resolution and is a highly effective antimicrobial treatment that does not easily induce drug resistance; it has also stimulated the scientific research enthusiasm of researchers and has become a research hotspot in the antimicrobial field. In this review, the photodynamic antibacterial applications of conjugated polymers with different structure types are summarized, and their development directions are considered.

## 1. Introduction

The rapid increase in antibiotic resistance among many species of pathogens has occurred mainly during the past 50 years, a period known as the “antibiotic age”. Bacteria replicate very rapidly, and a mutation that helps the organism survive in the presence of antibiotic drugs will soon dominate the entire microbial population [1,2]. Photodynamic therapy (PDT) was discovered more than 100 years ago by observing how harmless dyes and visible light killed microorganisms when combined in vitro. Since then, PDT has mainly been developed as a treatment for cancer, eye diseases, and microbe-related dermatosis [3,4]. In recent years, however, there has been a resurgence of interest in the antimicrobial effects of photodynamic therapy and its proposed use as a treatment for a variety of local infections. The emergence of this interest is largely driven by the significant increase in the multiple resistances of pathogens.

PDT mainly utilizes the strong light absorption capacity of a photosensitizer (PS). Under light irradiation, the photosensitizer can absorb light energy to sensitize the surrounding oxygen to produce reactive oxygen species (ROS) that are cytotoxic, including singlet oxygen (^1^O_2_), superoxide anion (•O_2_^−^), hydrogen peroxide (H_2_O_2_), hydroxyl radical (•OH), etc. [5,6,7]. ROS can destroy the cell membrane of the pathogens and cause a large amount of the intracellular content to flow out, followed by the killing of nearby pathogenic microorganisms. Currently, PDT is being considered as an effective alternative technology for microbial infection treatment, mainly due to the high responsiveness and short lifespan of ROS, which is a highly localized effect [8,9,10,11,12,13]. Compared with conventional antibiotic treatment, photodynamic antimicrobial therapy (PDAT) has the advantages of high selectivity for pathogenic destruction; it is non-invasive and has low side effects, and it is not prone to cause the development of resistance [14]. 

**Figure 1:**
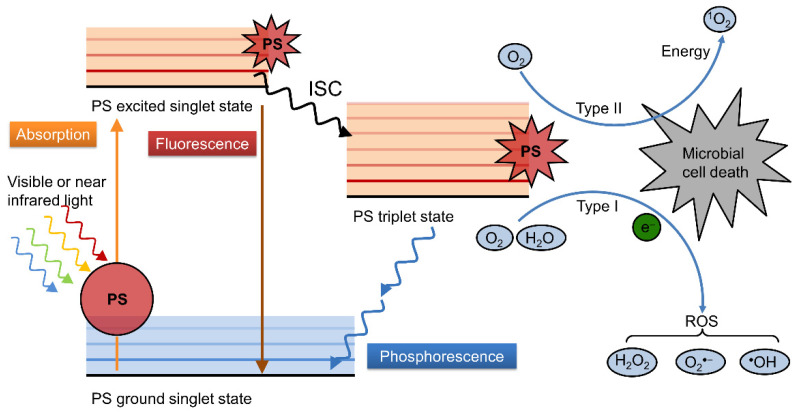
Illustration of photochemical process of PS in PDAT.

The photochemical process in PDAT to produce ROS is illustrated in Figure 1; the photosensitizer in the ground state (S_0_) absorbs energy and transitions to the excited state (S_1_, S_2_). Some of the excited PS molecules go back to the ground state in the form of radiative transition accompanied by the emission of fluorescence, and the other part jumps to the excited triple state (T) through the intersystem crossing (ISC) way. Then, there are two pathways for the PS in the T state to generate ROS. In the Type I way, the PS in the T state sensitizes the surrounding oxygen through electron transfer to produce H_2_O_2_, •O_2_^−^, and •OH, which has a low dependence on oxygen and is expected in a hypoxic environment. This process mainly occurs in the cell membrane, disrupting the integrity of the cell membrane and thereby increasing its permeability. In the Type II way, the PS in the T state transforms energy to triplet oxygen (^3^O_2_) to produce singlet oxygen (^1^O_2_), in which the ^1^O_2_ generation efficiency relies on the oxygen content, and the ^1^O_2_ can directly cause the peroxidation of the lipid of the cell membrane or oxidize the protein and nucleic acid to cause gene mutation and the inactivation of microbes. The type I and type II pathways are mutually competitive and mainly depend on the structural properties of the PS, the surrounding solvent environment and the oxygen concentration [5,15,16,17].

The photosensitizer is the key to determining the therapeutic effect of PDT. However, some typical photosensitizers, such as phthalocyanine and porphyrin, have a limited practical application due to their small absorption cross-sections and short-wavelength absorption. Compared with small molecule photosensitizers, conjugated polymers have long-range π-conjugated structures and strong light-capture abilities and photosensitization properties [18,19,20] and have a high ROS production efficiency upon light irradiation to effectively kill pathogenic microorganisms. Therefore, conjugated polymers have been successfully applied in the photodynamic treatment of microbial infections [21,22,23,24,25,26]. The structures of the antimicrobial conjugated polymer include, as has been reported, polythiophene, polyfluorene, poly(*p*-phenylene vinylene), and copolymers composed of different conjugated units.

## 2. Thiophene-Based Conjugated Polymers

**Figure 2:**
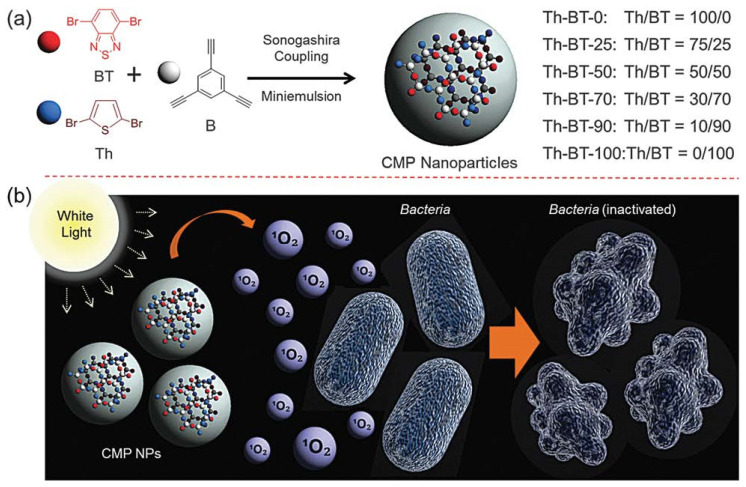
(**a**) Illustration of conjugated microporous polymer nanoparticles, route of synthesis, and proportion of monomers in the polymer skeleton. (**b**) Schematic diagram of bacterial inactivation mechanism of conjugated microporous polymer nanoparticles. Reproduced from Ref. [29] with permission from the Royal Society of Chemistry.

Thiophene is a heterocyclic compound as well as a sulfur ether. One of the two lone electron pairs of sulfur atoms is conjugated with two double bonds to form a delocalized π bond. Thiophene-based conjugated polymers have a flexible chain structure, which facilitates their interaction with pathogenic microorganisms [27,28]. In addition, due to the introduction of sulfur atoms, the absorption of thiophene-based conjugated polymers shifts to long wavelengths, thus facilitating the absorption of visible light for photodynamic therapy. Zhang et al. reported a series of thiophene-based conjugated microporous polymer nanoparticles and investigated the structure–activity relationship between conjugated microporous polymer nanoparticles doped with different proportions of electron-donating (thiophene) and electron-deficient (benzothiadiazole) groups and their antimicrobial activities [29]. As shown in Figure 2, the higher that the proportion of the electron-deficient group benzothiadiazole is doped in conjugated microporous polymer nanoparticles, the higher the HOMO-LOMO band gap and the lower the fluorescence intensity appears. By cyclic voltammetry, they found that the redox potential increased with the increase in the proportion of benzothiadiazole in the conjugated microporous polymer nanoparticles, and the redox potential was highest when the conjugated microporous polymer nanoparticles only contained the benzothiadiazole unit. A different doping ratio of benzothiadiazole in conjugated microporous polymer nanoparticles influenced the production of singlet oxygen. Along with the rising contents of the electron-deficient group in the polymer nanoparticles, the production of singlet oxygen increased. Finally, the antibacterial activity of these conjugated microporous polymer nanoparticles toward *E. coli* was assessed, and reactive oxygen radical scavenging experiments showed that the bactericidal activity of the polymer nanoparticles was attributed to the photosensitization, which generated singlet oxygen, which oxidized the lipid membrane to damage the cell membrane permeability of the bacteria. On the other hand, the polymer nanoparticles reacted directly with the bacterial lipid membrane to produce hydroxyl radicals that eventually killed the bacteria. This conclusion proves that thiophene-based conjugated microporous polymer nanoparticles have a promising application as a photodynamic therapy reagent.

**Figure 3:**
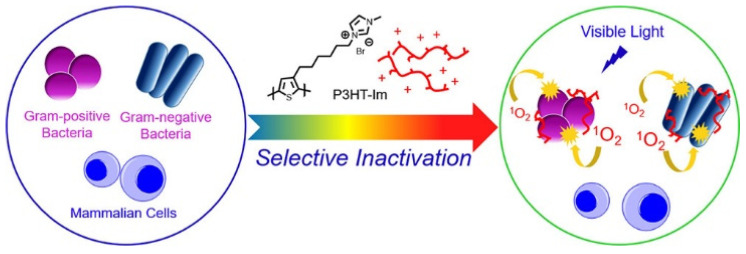
Schematic diagram of selective inactivation of bacteria over mammalian cells by P3HT-Im. Reprinted with permission from Ref. [30]. Copyright 2017 American Chemical Society.

Other than the preparation of nanoparticles to obtain hydrophilic conjugated polymer materials, the modification of the cationic group to the pendent of the conjugated polymer side chains is also an alternative strategy. Huang et al. reported a water-soluble polythiophene (P3HT-Im) derivative with side chains modified with positively charged methyl imidazole groups for the selective imaging and inactivation of bacteria on mammalian cells (Figure 3) [30]. On the one hand, due to the cationic group, P3HT-Im more easily absorbs to the negatively charged surface of the bacteria, which have more negative surface potentials than mammalian cells; therefore, a selective combination with bacteria rather than mammalian cells for P3HT-Im was observed. On the other hand, because of the tight binding effect between P3HT-Im and bacteria, the ROS production efficiency of P3HT-Im to photosensitize oxygen was enhanced. At a low concentration (0.1 μg/mL), P3HT-Im could effectively inactivate Gram-positive and Gram-negative bacteria at a small dose of visible light (8.2 J/cm^2^) but had no harmful effect on mammalian cells in achieving the purpose of the selective killing of pathogens.

**Figure 4:**
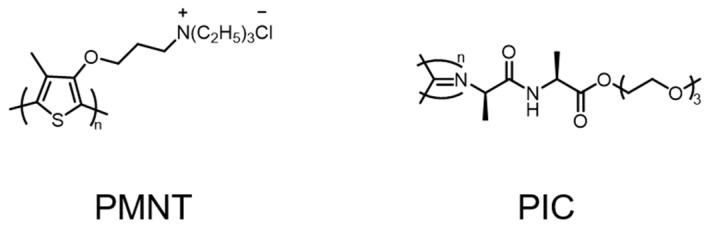
Chemical structures of PMNT and PIC.

As polythiophene derivatives are flexible and irregularly coiled, changing the irregularly coiled configuration of polythiophene can affect its optical properties and thus affect its photosensitization ability to generate ROS [31]. Xing et al. designed a conjugated polymer biomimetic fibrous polyisocyanide (PIC) hydrogel based on a water-soluble polythiophene (PMNT), as shown in Figure 4 [32]. The maximum absorption peak of PMNT itself was at 410 nm, and two new peaks appeared in the long wavelength range (589 nm and 543 nm) after adding PIC, proving that the PIC polymer acts as a scaffold to capture and line up the PMNT backbone into a highly ordered conformation, which results in red shifts and new sharp bands in the absorption and fluorescence spectra. A fluorescent probe was used to monitor the ROS production to show that the ROS production efficiency of this composite hydrogel under 600 nm light was much higher than that of the PMNT itself. In addition, it was also found that the ROS generation was easily controlled by irradiation time. Fluorescence microscopy showed that PMNT/PIC could target the microbial surface and effectively bind a variety of pathogens. Antimicrobial experiments further proved that the PMNT/PIC hybrid hydrogel showed effective photodynamic antimicrobial effects against Gram-negative bacteria, Gram-positive bacteria, and fungi, and the killing efficiency was significantly enhanced with the increase of the PMNT concentration in the hydrogel. The lowest inhibitory concentration of the PMNT in the hydrogel was 5.0 μM against *B. subtilis* and *C. albicans* and 10.0 μM against *E. coli*, which further demonstrated the broad spectrum and highly efficient photodynamic antimicrobial ability of the PMNT/PIC hybrid hydrogel. More importantly, PMNT/PIC hybrid hydrogels retain the strain-hardening properties of PIC itself, while combining its thermal reversibility and biocompatibility, and are expected to be used as intrinsic anti-infection wound dressings and provide a starting point for the development of other functional composite biomimetic materials.

**Figure 5:**
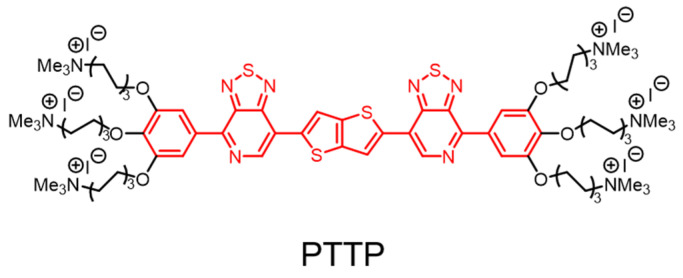
Structure of conjugated oligo-electrolyte PTTP.

Additionally, the thiophene unit is used as an electron-donating group to design conjugated polymers with excellent photophysical properties [33,34,35]. Bazan et al. designed and synthesized a membrane-embedded conjugated oligo-electrolyte (PTTP) (Figure 5) by introducing electron-deficient/electron-rich/electron-deficient units into the conjugated skeleton to regulate the bandgap to provide red-shifted absorption spectra [36]. The conjugated backbone of PTTP contains electron-rich thieno [3,2-b]thiophene, and the six quaternary ammonium groups modified at the end of the backbone increase the water solubility of PTTP and facilitate the electrostatic and hydrophobic interactions between PTTP and bacteria. It was found that the absorption wavelength reached the visible region with a maximum absorption of 507 nm, and the maximum emission was at 725 nm, which is favorable for PDAT and bacteria tracking. Upon the irradiation of white light, PTTP can effectively generate singlet oxygen in situ, and the efficiency of sensitizing the singlet oxygen is as high as 20%. PTTP is inserted into the bacterial cell membrane thanks to its appropriate molecular length. Accompanied by excellent sensitization ability, PTTP can quickly intercalate lipid membranes of bacteria and kill more than 99% of *E. coli* at an extremely low dose of light (0.6 J/cm^2^). This molecular design pattern provides guidance for the innovation of the conjugated oligomers with high photodynamic antimicrobial activity.

**Figure 6:**
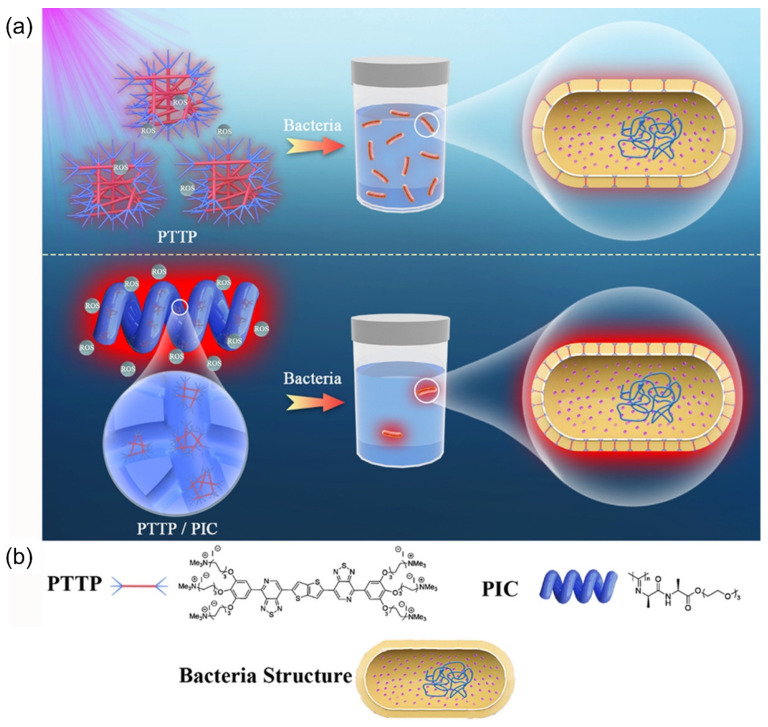
Illustration of PTTP itself and PIC/PTTP composite biomimetic hydrogel combined with pathogenic bacteria (**a**) and chemical structure of PTTP and PIC (**b**). Reprinted with permission from Ref. [37]. Copyright 2021 American Chemical Society.

Conjugated oligo-electrolytes (COEs) have good photodynamic antibacterial ability, excellent photostability, low cytotoxicity, and a low drug resistance tendency, which are ideal bactericidal components of biomimetic antibacterial hydrogels. However, the reactive oxygen species production efficiency and the antimicrobial activity of the COEs still need to be improved. Xing and Wang proposed a new method to prepare biomimetic antibacterial hydrogels by introducing PIC to regulate the aggregation of the COEs, with thiadiazolidine-thiophene-thiadiazolidine as the backbone (PTTP), so as to improve the antibacterial efficiency and biocompatibility [37]. The PIC/PTTP composite biomimetic hydrogel with high antibacterial efficiency was prepared. As shown in Figure 6, PTTP forms aggregates in aqueous solution, which are absorbed by bacteria and embedded in the membrane. The PIC hydrogel can regulate the aggregation behavior and aggregation size of PTTP in water and disperse them in the process of self-assembly by forming PTTP/PIC hydrogel, which can improve the ROS production efficiency of PTTP and the ability to bind to the bacteria, thus improving the antibacterial efficiency. The photodynamic killing effect on *E. coli* and *S. aureus* (91%) was much better than the PTTP itself (55%). Fortunately, extensive molecular structural exploration is not required to improve the photodynamic therapy activity of COEs, as it can be regulated by PIC to control the COE aggregation. The intermolecular interactions, such as the π-π interactions between the conjugated backbones during self-assembly, will lead to changes in the optical and electronic properties of COEs for biological applications. This method provides a simple and efficient strategy to improve the effect of the photodynamic therapy of water-soluble conjugated systems to design and prepare highly effective antibacterial hydrogels.

**Figure 7:**
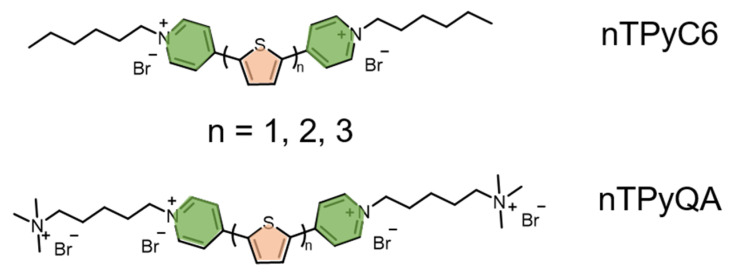
Structures of water-soluble thiophene derivatives for bacteria imaging and PDAT.

The introduction of heavy atoms can effectively promote the generation of a triplet state and improve the yield of ROS under irradiation [38,39,40]. Moreover, the addition of π-conjugated bridging units can effectively improve electron acceptances, reduce band gaps, and increase electron mobility. Guo et al. reported a series of water-soluble cationic thiophene derivative photosensitizers (nTPy-Rs) for photodynamic therapy (Figure 7) [41]. The UV-Vis absorption spectra showed that with the increase of the thiophene group number, the absorption wavelength of nTPy-Rs with the same functional scaffold was significantly red shifted, covering a wider visible region. The fluorescence spectra showed that the maximum emission wavelength of TPyC6 was 369 nm, while the maximum emission wavelengths of 2TPyC6 with two thiophene units and 3TPyC6 with three thiophene units were 449 nm and 467 nm, respectively, demonstrating an obvious red shift. nTPy-Rs have great potential for bacterial imaging due to their maximum fluorescence emission range of 430 to 600 nm, which is located in the visible region. All these results proved that with the increase of the thiophene group number, the molar extinction coefficient of the photosensitizer increased, and in the absorption and fluorescence spectra of the photosensitizer an obvious red shift occurred. Therefore, the extensive absorption in the visible light range lays a solid foundation for nTPy-Rs as effective photosensitizers. The introduction of the cationic pyridine group into the thiophene skeleton can enhance its solubility and improve its affinity with negatively charged bacteria through electrostatic interaction, thus effectively improving the bacterial imaging and antimicrobial activity. The photoelectric properties and ROS production ability of nTPy-Rs can be precisely regulated by adjusting the number of thiophene groups. Among nTPy-Rs, 2TPyC6 containing two thiophene rings and an alkyl side chain (C6) had the best bactericidal effect. The introduction of C6 significantly improved the efficiency of PDT, mainly due to the fact that C6 can form cavities in the cell membrane through hydrophobic action and thus more efficiently deliver ROS, leading to bacterial death. The MIC values of 2TPyC6 against *E. coli* and *S. aureus* were as low as 20 ng/mL and 4.5 ng/mL, respectively, and were the lowest concentrations of photosensitizer used for PDT inhibition to date. Confocal laser scanning microscopy (CLSM) showed that 2TPyC6 could be efficiently immobilized on bacterial membranes, rapidly enter bacteria, and accumulate on the genetic substance. Scanning electron microscopy (SEM) and transmission electron microscopy (TEM) demonstrated that the good antibacterial activity of 2TPyC6 mainly comes from the destruction of the membrane, which helps ROS to enter the bacteria and destroy the bacterial organelles, leading to final death. Therefore, this class of thiophene derivatives with extended absorption in the visible light range by regulating the number of thiophene groups has low cytotoxicity and excellent antimicrobial properties and has great potential for PDT drugs.

## 3. Fluorene-Based Conjugated Polymers

**Figure 8:**
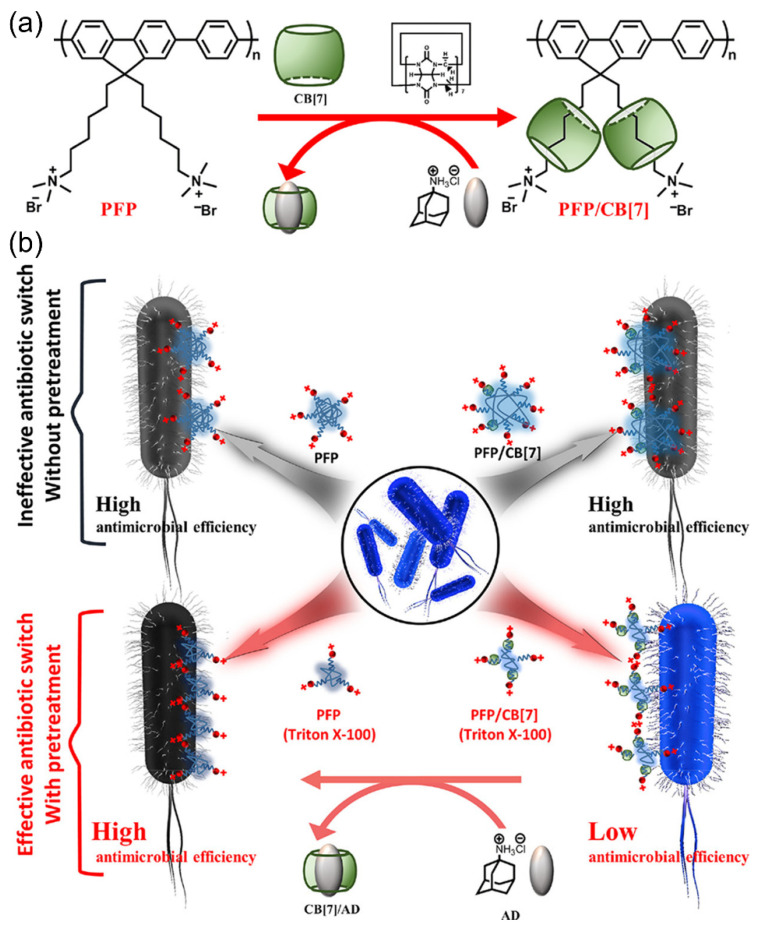
(**a**) The supramolecular assembly process between PFP and CB [7] and the de-assembly process of PFP/CB [7] complex after adding AD. (**b**) Schematic diagram of antimicrobial process of PFP/CB [7] before and after Triton X-100 pretreatment. Reprinted with permission from Ref. [49]. Copyright 2017 American Chemical Society.

Polyfluorene derivatives are one of the conjugated polymers synthesized and modified into cationic conjugated polymers at the early age of research of conjugated polymers for the biomedical applications. In the past 20 years, cationic polyfluorene derivatives have been widely used in biological applications due to their excellent optical properties and bio-affinity [42,43,44,45,46,47,48]. However, due to their rigid structure and aggregation behavior in water, their application in the antibacterial field is not ideal and the regulation of the antimicrobial activity of polyfluorene derivatives is expected. In 2017, Wang et al. reported an improved pretreatment strategy to obtain a more effective antibiotic switch based on a polyfluorene derivative (PFP) and a cucurbit [7] uril (CB [7]), which can regulate the antibacterial effect at any time with aid of a surfactant (Triton X-100) [49]. The switch can regulate the aggregation state of the PFP prior to the supramolecular self-assembly process, successfully achieving reversible control of the bactericidal activity of PFP under dark and white light irradiation (Figure 8). In a single polymer PFP solution, the amphiphilic structure will prompt the polymers to gather themselves together, and with the addition of CB [7] solution, the interaction of PFP and CB [7] in the space steric effect will reduce the aggregation degree of PFP, but CB [7] cannot effectively shield the quaternary ammonium salt groups of the side chains, leading to the lower shielding effect on the sterilization activity of the PFP. It was found that Triton X-100 could regulate the aggregation state of conjugated polymers. When Triton X-100 is first added to the PFP solution to reduce the aggregation degree, the subsequent addition of CB [7] can block the quaternary ammonium salt group of the polymer side chain and reduce the bactericidal activity (the antibacterial switch is turned off). After adding the competitive molecule amantadine (AD), with stronger affinity to CB [7], into the PFP/CB [7] complex, a more stable CB [7]/AD will be formed, and the quaternary ammonium salt group will be released to enhance the bactericidal activity (the antibacterial switch is turned on). This approach would not only reduce the overuse of antibiotics but also reduce environmental accumulation. This pretreatment strategy can be applied to a variety of polymers with similar properties, which will be of great help in the regulation of antimicrobial materials.

**Figure 9:**
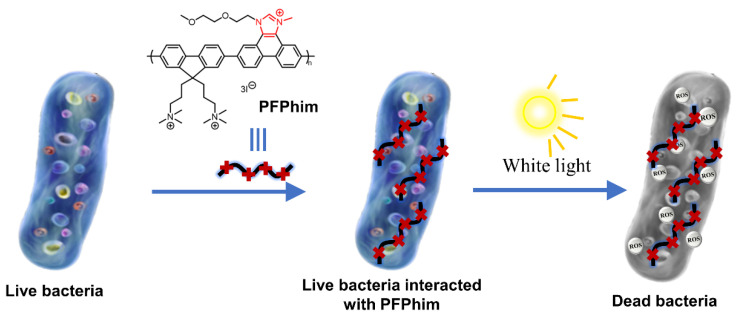
Schematic diagram of PFPhim structure and the inhibition of *E. coli* via PDT. Reprinted with permission from Ref. [50]. Copyright 2019, Elsevier B.V.

Yoon et al. developed a cationic skeleton conjugated polymer based on polyfluorene (PFPhim) for antimicrobial use. In addition to the quaternary ammonium groups in the side chain, PFPhim also contains a cationic imidazonium group in its conjugated skeleton (Figure 9) [50]. The cationic skeleton is able to produce stronger interactions with negatively charged bacterial membranes and to enhance antimicrobial activity under dark and light conditions. The experimental results showed that the PFPhim with the positively charged skeleton exhibited stronger antibacterial ability than the conjugated polymer containing a neutral backbone. Zeta potential proved that positively charged PFPhim could effectively adhere to the bacterial membrane of pathogenic bacteria through electrostatic interaction, and SEM observation showed that pathogenic bacteria would aggregate after binding to PFPhim with the surface collapse and rupture. All these phenomena reflected that PFPhim would cause serious damage to the bacterial membrane. The ROS probe proved that PFPhim had excellent ROS production ability under light. PFPhim at a concentration of 16 μM could kill about 70% of *E. coli* in the dark. Under white light irradiation (20 mW/cm^2^), PFPhim sensitized the surrounding oxygen to produce ROS; so, the antibacterial rate was up to 94.7% at the same concentration. Meanwhile, SEM observation also showed that the bacterial membrane was more seriously damaged under light, indicating that PFPhim had good dark and photo-toxicity for bacteria. At the same time, standard MTT also demonstrated that PFPhim had almost no cytotoxicity at the experimental concentration. Therefore, PFPhim can effectively bind pathogens through its positive charge and generate ROS under light to improve its antibacterial efficiency and is an excellent photodynamic antimicrobial agent.

**Figure 10:**
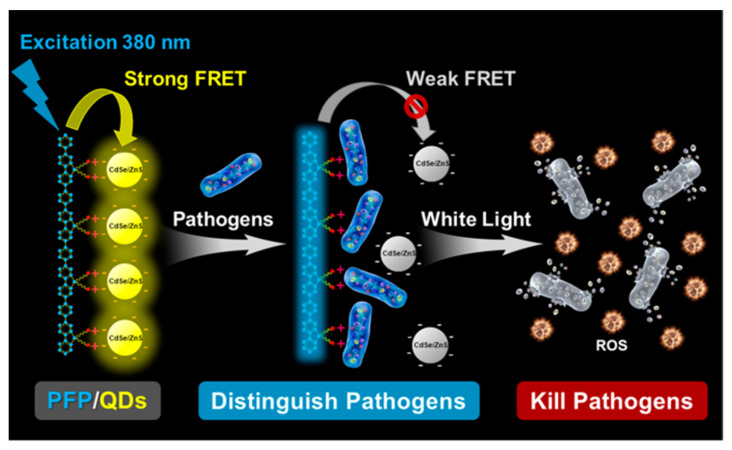
Schematic diagram of PFP/QDs hybrid material for pathogen identification and killing. Reprinted with permission from Ref. [30]. Copyright 2020 American Chemical Society.

Conjugated-polymer-based multi-functional systems for simultaneously achieving microbe detection and killing are also desired. Wang et al. designed and constructed a novel pathogen recognition and killing platform based on PFP/quantum dots (QDs) hybrid materials through fluorescence resonance energy transfer (FRET) technology [51]. The hybrid material (Figure 10) consists of a water-soluble anionic CdSe/ZnS QDs and a cationic polyfluorene derivative (PFP) through electrostatic interaction, thereby improving the effective FRET between the PFP and the QDs. After the addition of different pathogen strains, the FRET from the PFP to the QDs is interrupted due to competitive binding between the PFP and the pathogens, and different species of pathogens can induce various FRET destruction; so, the pathogens can be recognized by different fluorescence response signals. The fluorescence intensity changes measured from different channels are processed by linear discriminant analysis (LDA) to provide an effective and accurate pathogen classification. Taking advantage of the phototoxicity of PFP, which sensitizes oxygen to produce a large amount of ROS under light irradiation, the PFP/QDs kill pathogenic bacteria when exposed to light. The complexation of PFP and QDs also reduces the dark toxicity to a more desirable level; so, it is possible to achieve controlled killing of pathogens. This strategy provides a promising platform for pathogenic infection diagnosis and treatment based on PFP, QDs, FRET, and phototoxicity.

Cheng et al. reported a polymyxin B-modified conjugated oligomer nanoparticle (PMB-CON) based on fluorene and thiophene units for targeting photodynamic killing-resistant bacteria [52]. Nanoparticles can selectively identify resistant Gram-negative bacteria over Gram-positive bacteria and fungi. At the concentration of 0.8 μg/mL, the photokilling efficiency of PMB-CON against Gram-negative bacteria reached 99%, whereas the unmodified nanoparticles could only kill ~30% of the bacteria. In addition, PMB-CON showed photodynamic killing activity only against Gram-negative bacteria, rather than Gram-positive bacteria and fungi, due to the targeting effect of polymyxin B. This study expands the application of antibiotics and opens a new way for the enhancement of photodynamic antimicrobial therapy and for the combating of bacterial resistance.

Jiang et al. designed an optical probe based on a fluorene unit and an iridium complex for hypoxia imaging and photodynamic antibacterial therapy [53]. The probe has a good singlet oxygen generation ability under light irradiation and can effectively respond to low oxygen concentration in a hypoxic environment in photodynamic antimicrobial therapy. After 5 min of light exposure, the probe inhibited 58% of *S. aureus*. In addition, the probe was further cross-linked with carboxymethyl chitosan (CMCS) and sodium alginate (SA) to form a CSGI hydrogel. The hydrogel can effectively inhibit the bacterial growth of wounds and promote the healing of chronic wounds in diabetic mice by generating singlet oxygen under light and has potential clinical application prospects.

## 4. Poly/oligo(*p*-phenylene vinylene)-Based Polymers

**Figure 11:**
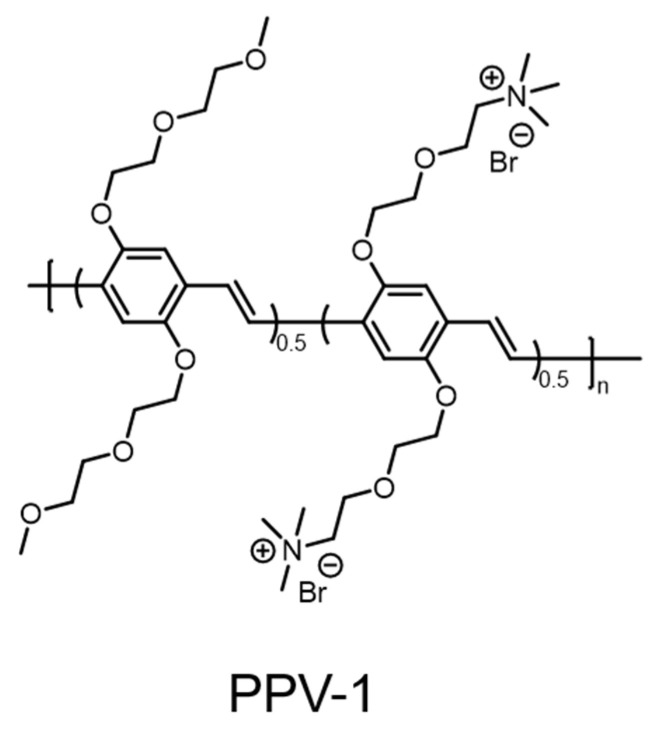
Chemical structure of PPV-1 for selective recognition, imaging, and killing of bacteria over mammalian cells.

The first multifunctional conjugated polymer based on poly(*p*-phenylene vinylene) (PPV) was synthesized by Zhu et al.; it combines the functions of selective recognition, imaging, and the killing of bacteria over mammalian cells into a single system (Figure 11) [54]. The system is mainly composed of a poly(*p*-phenylene vinylene) backbone with positively charged oligoethylene glycol side chains (PPV-1). Because the bacterial surface has a higher negative charge density than mammalian cells and the oligoethylene glycol group of PPV-1 prevents its non-specific adsorption on the surface of mammalian cells, PPV-1 can selectively bind to bacteria rather than mammalian cells through electrostatic interaction. The bright green fluorescence enables PPV-1 to selectively image bacteria by effectively avoiding the labeling step of conventional imaging. Meanwhile, with exposure to white light (400–800 nm), PPV-1 can produce ROS capable of killing bacteria at a light dose of 27 J/cm^2^. The development of this polymer had a profound impact on the subsequent exploration of antimicrobial materials and the potential applications in the selective pathogen killing of conjugated polymer.

**Figure 12:**
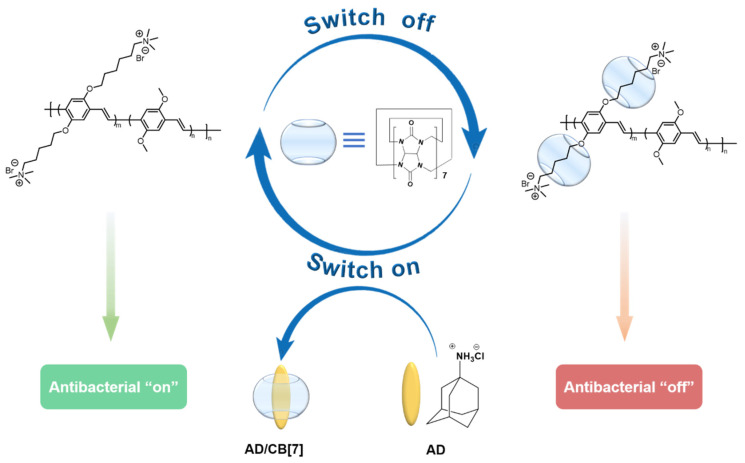
The supramolecular assembly process and the disassembly process of PPV/CB [7] complex.

In order to combat bacterial resistance and avoid the long-term exposure of antimicrobial materials to pathogens for the development of resistance, Bai et al. developed a supramolecular antimicrobial system based on a PPV derivative for the first time; it uses the molecules of CB [7] and AD to turn on or off the antimicrobial activity of PPV itself [55]. As shown in Figure 12, cationic PPV can form a non-covalent complex with CB [7], which has a hydrophilic exterior and hydrophobic cavity for the encapsulation of the quaternary ammonium (QA) salt group; the PPV/CB [7] complex turns off the antibacterial activity of PPV. The addition of AD results in the formation of a more stable CB [7]/AD complex through the competition to release PPV, which restores its antimicrobial activity. PPV can be inserted into *E. coli* through a QA side chain to effectively inhibit its growth, and the antibacterial rate can reach 70%. The inhibitory efficiency of the PPV/CB [7] complex decreased to 30%. By introducing and forming the CB [7]/AD complex with AD, the release of QA groups and the recovery of the antibacterial ability of the system are promoted. At the same time, it was confirmed that under white light irradiation the generation of ROS made the material have higher antibacterial activity. This simple and effective strategy does not require any chemical modification of the active site of the antimicrobial agent and can also modulate the antimicrobial activity of classical antibiotics or photosensitizers in photodynamic therapy. The “antibiotic switch”, as one of the most promising drug candidates, has been shown to reversibly control antimicrobial activity through supramolecular assembly/disassembly between cationic conjugated polymer and CB [7], which implies that we might prevent and delay the resistance development of pathogens.

**Figure 13:**
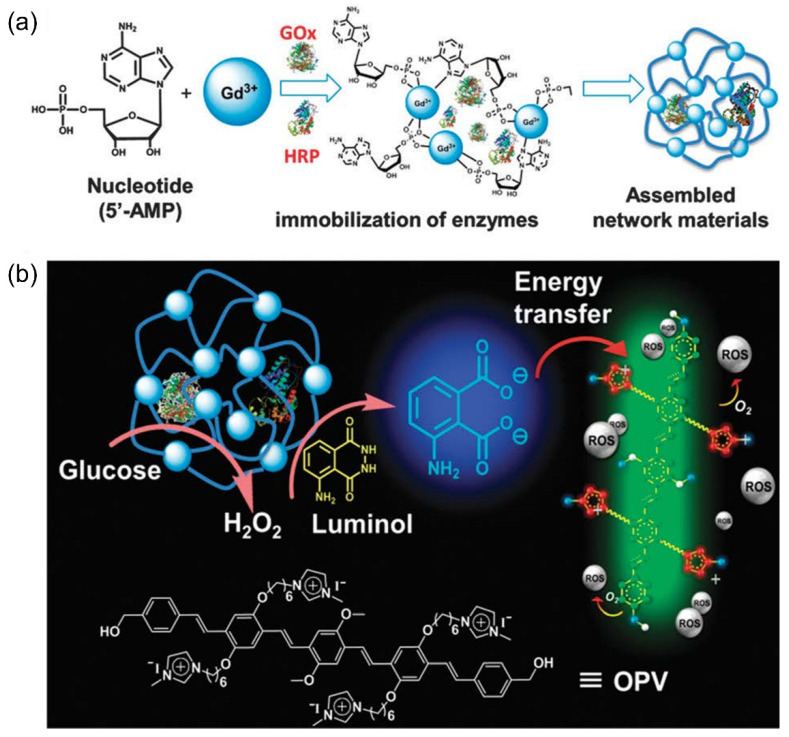
(**a**) Schematic diagram of the preparation of networks containing GOx and HRP. (**b**) Illustration of a glucose-activated BRET system for antimicrobial application without external light source. Reproduced from Ref. [57] with permission from the Royal Society of Chemistry.

The light absorption and the scattering effect of biological tissue prevent the external light from penetrating the deep tissue; so, the requirement of an external light source in PDAT limits its effective application in deep tissue infections. Therefore, it is of great significance to develop a new PDAT method that is independent of an external light source. Yuan et al. pioneered the bioluminescence resonance energy transfer (BRET) system based on oligo(*p*-phenylene vinylene) (OPV) and luminol luminescence for photodynamic antimicrobial applications for the first time [56,57]. As shown in Figure 13, the BRET system sensitizes oxygen to produce ROS using chemical molecules rather than external light irradiation. They prepared a network encapsulating glucose oxidase (GOx) and horseradish peroxidase (HRP) and GOx catalyzed glucose to form hydrogen peroxide with the subsequent catalytic decomposition by HRP, followed by the oxidizing of luminol to emit blue light at 425 nm, which could be absorbed by OPV to generate ROS, thus triggering the PDAT system. The immobilization of the two enzymes in the networks can limit their diffusion in the solution and improve the efficiency of the overall enzyme cascade reaction. More importantly, glucose is abundant in the tissue to induce the enzymatic cascade; so, the whole process can be conducted with just the addition of luminol. This BRET system inhibited *E. coli* by up to 80% and *C. albicans* by up to 70%, showing broad-spectrum antimicrobial activity. This is a new photodynamic antimicrobial system that can replace light and use glucose to fight against bacteria and fungi effectively. At the same time, it can solve the cytotoxic problem of adding hydrogen peroxide directly and can open up a new treatment mode for pathogenic infections.

**Figure 14:**
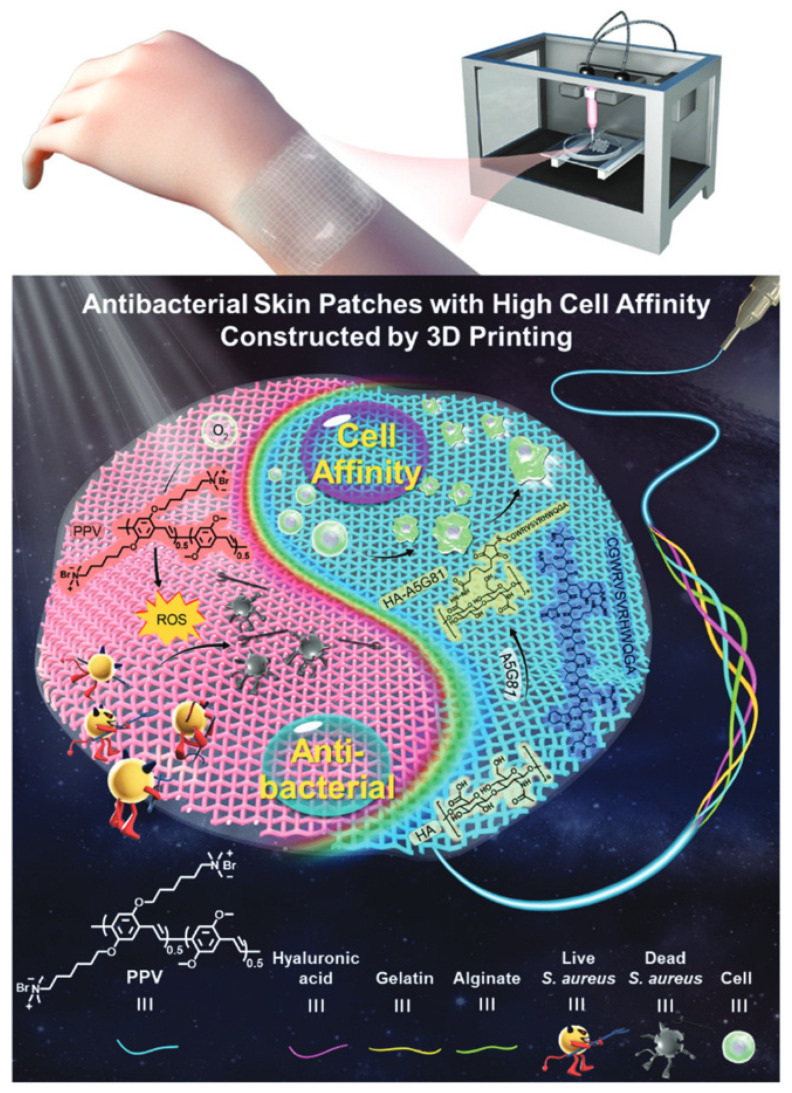
Illustration of 3D printed high-cell affinity enhanced antibacterial patch for wound repair based on conjugated polymer inks. Reproduced from Ref. [58] with permission from the Royal Society of Chemistry.

Cationic conjugated polymer can form a uniform system with anionic hydrogel through electrostatic interaction, and the electrostatic interactions between them can also inhibit the out-diffusion of the conjugated polymer and reduce the influence on the surrounding tissues, which could be one of the important functional materials for the construction of artificial antibacterial skin. Zhao et al. utilized a gelatin/sodium alginate (Gel/Alg) bio-ink containing cationic PPV to construct artificial antibacterial skin with high cell affinity through 3D bioprinting technology as shown in Figure 14 [58]. The antibacterial skin showed a good antibacterial effect both in a solution and in a rat model. When the concentration of PPV in Gel/Alg hydrogel was 200 μg/mL, the photodynamic inhibition rate was 73%. When the PPV concentration increased to 300 μg/mL, the PPV/Gel/Alg hydrogel could achieve more than 90% of the photodynamic bacteria damage. There was no obvious dark toxicity at a high concentration of PPV, which further indicated the good biocompatibility of PPV/Gel/Alg hydrogel. Wound healing in a rat experiment indicated that the PPV/Gel/Alg hydrogel made the infectious wound obviously callused and narrow under light irradiation, but the wounds in the control groups displayed a suppuration and tissue fluid leakage. After dilution of the wound tissue fluid, it was found that there were almost no bacteria in the wound treated with PPV/Gel/Alg hydrogel in the presence of light, whereas the bacteria number in the control groups was very high, indicating that PPV artificial skin has good antibacterial properties in vivo. The successful construction of artificial antibacterial skin with high bio-affinity based on PPV provides a referable idea for 3D bioprinting technology to fabricate tissues/organs in vitro.

## 5. Conjugated Copolymers

**Figure 15:**
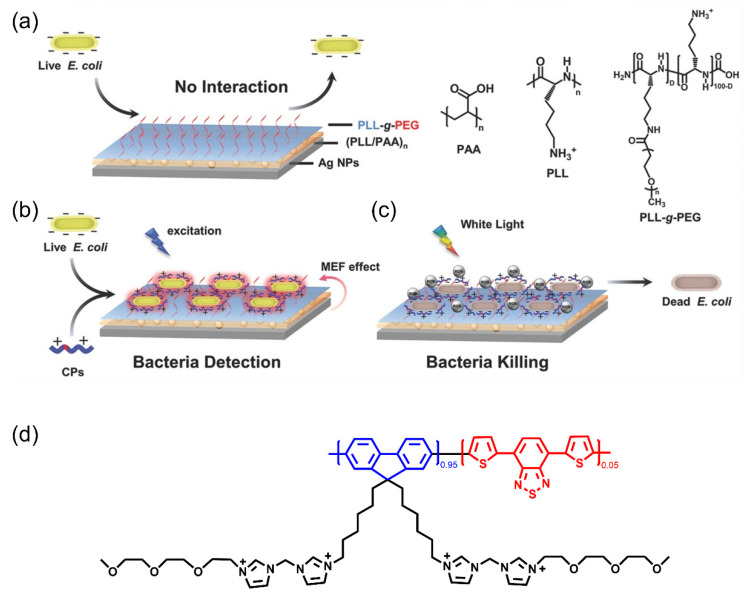
Illustration of multifunctional platform based on conjugated polymer–silver nanostructure for detecting and killing bacteria. (**a**) The anti-adsorption surface based on silver nanostructure. (**b**) Bacteria detection and killing surface modified with conjugated polymer. (**c**) CPs produced ROS under white light irradiation for disinfection. (**d**) Chemical structures of cationic PFDBT-BIMEG. Reproduced from Ref. [59] with permission from the Royal Society of Chemistry.

Applying different conjugated units to synthesize copolymers could flexibly modulate the photophysical properties and functions of conjugated polymers. Li et al. synthesized a conjugated polymer through copolymerizing fluorene and thiophene units (PFDBT-BIMEG) and developed a multifunctional platform based on a conjugated polymer–silver nanostructure pair, which can be used to detect and kill bacteria (Figure 15) [59]. The metal-enhanced fluorescence (MEF) effect of silver nanostructures enhances the fluorescence intensity of PFDBT-BIMEG. The conjugated backbone and hydrophilic side chains of cationic PFDBT-BIMEG enable it to capture *E. coli* on the surface of the platform, thereby exciting PFDBT-BIMEG to generate fluorescence signals in response to binding to bacteria. Additionally, the MEF effect of silver nanoparticles can enhance the fluorescence intensity of PFDBT-BIMEG to achieve a highly sensitive detection of bacteria. In addition, PFDBT-BIMEG is an efficient antibacterial system due to ROS production under light. The platform can not only realize the detection of bacteria, it also has antibacterial activity, which provides inspiration for the design of surface antibacterial materials.

**Figure 16:**
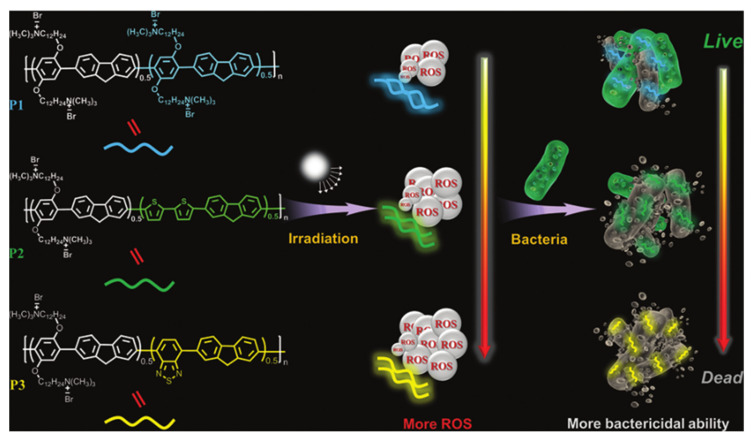
Schematic diagram of chemical structure and antimicrobial activity of P1-P3. Reproduced from Ref. [60] with permission from the Royal Society of Chemistry.

Wang et al. synthesized a series of biocompatible conjugated polymers by copolymerizing fluorene, thiophene, or benzene with adjustable bactericidal activity [60]. It was found that changing the backbone structure of the polymers could modulate the ROS generation capacity, and the donor–acceptor interaction had a significant impact on the performance of these conjugated polymers. Three conjugated polymers (P1, P2, P3) were obtained by combining fluorene with benzene, fluorene with thiophene, and fluorene with benzothiazole, respectively. P3 had the best ROS generation ability and optical stability (Figure 16). When exposed to white light (25 mW/cm^2^) for 15 min, the inhibition rate of ampicillin resistant *E. coli* was 97% at 3 µM of P3 and 100% at 5 µM. ROS produced by polymers can cause bacterial membrane damage and thus kill bacteria. After 5 days of treatment with P3 under light conditions, the wounds of mice were almost healed, which was consistent with the results of in vitro experiments. The experimental results of wound infection treatment in mice also showed that P3 had the best therapeutic effect. This study not only provides new fungicides for the practical treatment of infectious wounds, but also provides guidance for the design and structure regulation of photoactive fungicides.

Tang et al. designed and synthesized a series of polyphenylacetylene derivatives (PPE) with phenylacetylene and tetraphenylethene units [61]. The tetraphenylethene unit in the polymer endows the polymer with an aggregation-induced emission property to avoid self-quenching during imaging, which makes it beneficial for in vivo imaging. The obtained PPE can preferentially image bacteria rather than mammalian cells after being modified with the quaternary ammonium group in the side chains. PPE with quaternary ammonium pendants could kill more than 99% of *S. aureus* and drug-resistant bacteria at a low concentration of 5 μg/mL, with exposure under light for 10 min, demonstrating the efficient photodynamic antibacterial ability of PPE. This work provides a viable design strategy for the development of functional conjugated polymers with photodynamic antimicrobial capabilities to combat multidrug resistance.

**Figure 17:**
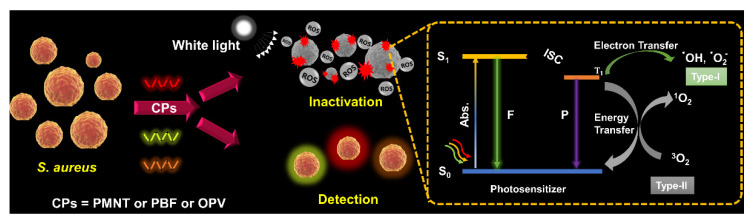
Schematic diagram of PMNT, PBF, and OPV for diagnosis and photodynamic killing of *S. aureus*. Reprinted with permission from Ref. [62]. Copyright 2022 American Chemical Society.

Yuan et al. evaluated and compared the antibacterial activity of three cationic conjugated materials with different backbones (thiophene-based PMNT, fluorene-coupled BODIPY-based PBF, and oligo(*p*-phenylene vinylene)-based OPV) [62]. The three conjugated polymers interact with pathogenic microorganisms through hydrophobic interaction and electrostatic interaction to achieve a photodynamic antibacterial effect under white light irradiation, and imaging of the pathogens can also be achieved (Figure 17). The results showed that OPV produced ROS mainly through the Type I process, which was more conducive to the treatment of drug-resistant bacterial infections in a hypoxic environment and could kill more than 98% of pathogenic *S. aureus* at a low concentration of 0.5 μM with white light irradiation. PBF (1 μM) was irradiated with white light (65 mW/cm^2^) for 10 min to reach the same antibacterial efficiency in the pathway of Type II. By changing the backbone structure of the conjugated polymer, the bactericidal ability can be adjusted to achieve an antibacterial effect at a low concentration. At the same time, due to their excellent fluorescence properties, these polymers can rapidly detect pathogenic microorganisms within 30 min. Therefore, cationic conjugated polymers with different skeleton structures have important value and application prospects for the integrating diagnosis and treatment of pathogenic infections under different oxygen content conditions.

## 6. Summary and Outlook

Conjugated polymers are widely used in the field of biomedicine because of their excellent light absorption, high fluorescence quantum yield, and photostability. Positively charged conjugated polymers (including oligomers) can be combined with pathogens by electrostatic and hydrophobic interactions, followed by the effective killing of the pathogenic microbes under the condition of light irradiation in which conjugated polymers can sensitize the surrounding oxygen to produce ROS. Although organic conjugated polymers have achieved remarkable results as antimicrobial materials, there are still some limitations that need to be further investigated and resolved. Firstly, PDAT needs to generate ROS under light irradiation to realize an antimicrobial effect, and photosensitizers must reach a sufficient distance from biological tissues to ensure the effectiveness of ROS; so, this passive excitation mode is not significant for the treatment of pathogenic infections in the deep tissue. Secondly, the development of conjugated polymers/oligomers with long wavelength absorption is a challenge and of great help for the treatment of deep tissue infections. Thirdly, most of the current research on antimicrobial polymer materials has been carried out in ordinary mouse models and still has a large gap compared with humans in a clinic. Therefore, the in vitro model cannot completely simulate the in vivo conditions, nor can it accurately replicate the in vivo cell–cell interactions. Finally, the mechanisms of action of the antimicrobial materials are also different in the dark and under light irradiation, and the long-term biosafety, degradation mechanisms, and metabolic pathways of conjugated polymers are the terms to be investigated. Furthermore, the design of low oxygen-dependence and dose-controllable photosensitizers is a research hotspot in the application of PDAT to solve clinical infections.

In conclusion, in the design of novel conjugated polymer or nanoparticle therapy systems, the development of multifunctional conjugated polymer therapy methods that integrate efficient phototherapy, intelligent response, targeting, and biodegradability will have important application prospects. In the future, antimicrobial materials that can trigger multiple therapeutic modes (photothermal, photodynamic, photocatalytic, etc.) with rapid metabolization by the living body need to be designed and synthesized, and their metabolic mechanisms need to be studied in detail. In addition, the simple synthesis and mass production of antimicrobial materials are favorable and better in in vivo and practical applications, thus greatly improving the efficiency of the clinical treatment of drug-resistant pathogenic infections.
